# Determinants of Adherence to Healthy Eating Patterns in a Population of Children and Adolescents: Evidence on the Mediterranean Diet in the City of Mataró (Catalonia, Spain)

**DOI:** 10.3390/nu11040854

**Published:** 2019-04-15

**Authors:** Ana Maria Arcila-Agudelo, Carmen Ferrer-Svoboda, Teresa Torres-Fernàndez, Andreu Farran-Codina

**Affiliations:** 1 Department of Nutrition, Food Science, and Gastronomy, XaRTA – INSA, Faculty of Pharmacy, University of Barcelona, Campus de l’Alimentació de Torribera, Av. Prat de la Riba, 171, Santa Coloma de Gramenet, E-08921 Barcelona, Spain; anamariaarcila1@gmail.com; 2 Blanquerna Faculty of Health Science, University Ramon Llull Carrer Padilla, 326-332, 08025 Barcelona, Spain; carmenfs@blanquerna.url.edu; 3 Social Welfare, Health and Consumption of the Mataró Town Hall, Avinguda de Puig i Cadafalch 101-111, 1er pis, 08303 Mataró, Spain; ttorres@ajmataro.cat

**Keywords:** healthy eating patterns, Mediterranean diet, children, adolescents, KIDMED

## Abstract

Despite its benefits, the Mediterranean diet (MD) is being abandoned or not adopted by young generations in most Mediterranean countries. In Spain, up to 69% of the child and adolescent population has been found to have suboptimal adherence to the MD. The aim of this study was to analyze which factors are associated with an optimal adherence to the MD in school-age children and adolescents from Mataró, Spain. A cross-sectional study was performed on 1177 children and adolescents aged between 6 and 18 years from Mataró. The Mediterranean Diet Quality Index for Children and Adolescents (KIDMED index) was used to evaluate adherence to a MD. We found that over 59% of subjects showed suboptimal adherence to a MD, with this prevalence being higher for secondary school than for primary school children. The factors positively associated with following an optimal MD were the mother’s education level, children at the primary school level, the absence of distractions at breakfast, and regular physical activity. The availability of spending money was negatively associated with the likelihood of optimal adherence to a MD. Future research should study more in-depth the possible causality between the factors studied and adherence to a MD.

## 1. Introduction

Globalization has led to a rise in the so-called “Western” dietary pattern, characterized by the presence of foods with high quantities of refined carbohydrates, sugars, salt, saturated fats, trans fats, animal proteins, and artificial coloring and flavoring [1]. In contrast to this trend, there remain other traditional dietary models considered highly beneficial to health, such as the Mediterranean diet (MD). This diet has been defined as a combination of dietary habits along with several sociocultural elements of the populations of the countries in the Mediterranean region [1,2,3]. In this dietary pattern, the consumption of cereals, legumes, nuts, fish, and olive oil predominate, and there is a low intake of red meat and processed foods. Other elements of interest linked to the MD include frugality and moderate to high levels of physical activity [3].

Multiple studies have suggested that a high degree of adherence to the MD is associated with a lower risk of several types of chronic and degenerative diseases, in turn increasing life expectancy and quality of life [4,5,6,7,8,9,10]. Despite these benefits, the MD is being abandoned or not adopted by young generations in most Mediterranean countries [11,12,13,14]. In Spain, up to 69% of the child and adolescent population has been found to have suboptimal adherence to the MD [11,15,16]. Similar percentages have been found in this segment of the population in countries such as Greece and Turkey [5,11,17]. In a recent study from northern Italy, an even bigger result was found, with over 80% of school-age children and adolescents having a suboptimal adherence to the MD [18]. This phenomenon has created a “nutritional transition”, where problems such as being overweight, obesity, and diet-related chronic diseases have become new challenges for public health systems in Mediterranean countries [13].

Under this scenario, understanding the factors associated with adherence to healthy dietary patterns and lifestyles in a school-age population is essential for an appropriate, focused design of public health interventions that will contribute to early adoption of healthy habits to reduce the impact of this nutritional transition [19,20,21]. Among the different factors studied for their possible association with whether or not children and adolescents follow a healthy diet have been (i) socioeconomic and demographic factors such as family composition, financial income, and parents’ education [16,17,22,23,24,25]; (ii) participation in regular physical activity [20,26,27,28]; and (iii) participation in sedentary activities both at school and outside of school [17,29,30,31]. As most of these results have focused on adolescents rather than children, there has been a lack of studies that have simultaneously investigated both populations, children and adolescents, and which factors might be associated with their adherence to the MD [32,33]. Thus, this study aimed to analyze the main common determinants of an optimal MD adherence in children and adolescents from Mataró, one of the largest cities in Catalonia, Spain.

## 2. Materials and Methods

### 2.1. Participants and Sampling

This study was carried out in the city of Mataró, which is a coastal city located near Barcelona (25 km) in Catalonia, Spain. The city has experienced an important increase in population in the last 50 years (from 40,407 inhabitants in 1960 to 122,905 in 2010) due to migration from other parts of Spain and, in recent years, from other nations (mainly from Morocco). Nowadays, 16.9% of the population is of foreign origin. The economy of Mataró is mainly based on services (63% of total invoicing) and industry (31%) [34]. Populations in the Mediterranean region have experienced an intense urbanization in the second half of the 20th century, and now two-thirds of the Mediterranean population are living in urban areas (>10,000 inhabitants) [35]. Based on the number of inhabitants, Mataró could be considered an average Mediterranean city.

The study population included children and adolescents aged between 6 and 18 years in 2011 from educational institutions in Mataró. Education centers in the city could be state schools (full public financing) or state-sanctioned private schools (mixed public and private financing). In total, there were 42 schools (64.3% public, 35.7% mixed) distributed into three categories and types: (i) Those with only a primary education level (17 public, 1 mixed); (ii) those with a primary and secondary education level (3 public, 12 mixed); and, (iii) those with only secondary school (7 public, 2 mixed). To obtain a representative sample of this population, we used a stratified random sampling method where the strata were the school type (i.e., public and mixed), within which we assumed that the students were a homogeneous group. First, from the universe of institutions, 18 schools were randomly chosen (43% of total schools), from which 11 were public (61.1%) and 7 were mixed (38.9%). Only two schools rejected participation in the study, and they were replaced by other randomly selected schools. According to the type of school, we interviewed 13 institutions with primary level education (40%) and 12 institutions with a secondary level (50%). Then, random samples were obtained for each stratum. In total, 668 primary school students aged 6 to 12 and 509 secondary school students aged 12 to 18 were interviewed, giving a total of 1177 children and adolescents from all of the schools sampled in the city. This sample of 17% of the total school population (*n* = 7045) constituted a representative sample of the population, with a 95% confidence interval and a margin of error of 2.66%. Data collection took place between January and October 2011. Sampling weights were not used because the sampling design was a complete randomness sample where all individuals had the same probability of being selected.

All subjects gave their informed consent for inclusion before they participated in the study. The study was conducted in accordance with the Declaration of Helsinki, and the protocol was approved by the Bioethics Committee of the University of Barcelona (IRB00003099).

### 2.2. Data Collection Instruments

To assess adherence to the MD, we used the Mediterranean Diet Quality Index for Children and Adolescents (KIDMED index), which is designed to assess this construct in a population of children and young people aged 2 to 24 years [16]. This index is determined from a 16-point questionnaire that assesses various dietary habits. Each answer is scored according to whether or not it is consistent with habits associated with the MD pattern, and scores are added up to demonstrate the total index of the subject’s adherence to the MD. The KIDMED index ranges from −4 (no adherence to the MD) to 12 (complete adherence to the MD). This index is then used to classify subjects into three categories according to their adherence to the MD. Originally, these categories were “very low” adherence to the MD (−4 to 3 points), “need to improve” (4 to 7 points), and “optimal” (8 to 12 points). In order to evaluate additional dimensions related to dietary habits and lifestyle, we applied a questionnaire based on the enKid study [36]. The intensity and type of physical activity was assessed using selected questions from the 1992–2003 Nutritional Survey of the Catalan Population (ENCAT) [37]. The section on socioeconomic factors was based on the Family Affluence Scale (FAS) [38,39]. Basic anthropometric data were collected by following the International Standards for Anthropometric Assessment (ISAK) protocol [40]. Participants’ weight was determined by using a Seca brand digital flat scale (Model 813) with a capacity of up to 200 kg and a precision of 100 g. Height was determined using the Seca stadiometer for mobile height measurement (Model 217), which has a maximum capacity of 205 cm and a precision of 1 mm (Seca GmbH & co., Hamburg, Germany).

The anthropometric variables were weight and height, from which the body mass index (BMI) was calculated (weight in kilograms divided by the square of height in meters). In order to estimate the *z*-score (a measure of how many standard deviations below or above the population mean a raw data point is), we used validated reference tables elaborated for Spain [41]. The sociodemographic variables used were sex, age, school level, parents’ educational level, parents’ place of birth, and availability of spending money at school (yes/no). The variables related to dietary habits were the presence of distractions during mealtimes, eating meals with company, and the presence of vending machines in school. The variables related to other habits and lifestyles were adequate hours of sleep according to the World Health Organization (WHO) (10 h for children and 8 h for adolescents) and frequency of physical activity per week, with a distinction between light and vigorous physical activity. All habits and lifestyle variables were treated as dichotomous variables (yes/no).

### 2.3. Data Collection Procedure

The schools selected in the random sampling were contacted, and an appointment was made with the principal or educational coordinator in each school to explain the study aims and requirements. We also gave an informative talk aimed at the students in the higher grades to encourage them to take part in the study and give them the chance to ask questions. In addition, each student in the sample was given an explanatory letter about the study and an informed consent form. For primary school, children took the consent form with them in order to have it signed by their parent/guardian, and they returned them within the specified time frame. In the case of high school students, the consent was signed by them inside the classroom and by their parents at home, and then was returned in printed format. Regarding the questionnaires, children from primary school took them in printed format, and then they were completed by their parents at home and returned within the corresponding period. The questionnaires were completed by the mother (72%), by both parents (10%), or by the father (9%). The high school students completed the questionnaires themselves inside the classroom, always helped by a senior researcher or a student of Human Nutrition and Dietetics or Physiotherapy, who were available to address doubts.

The data from the questionnaire and anthropometric tests were collected by a group of students in the later years of their studies for their Human Nutrition and Dietetics and Physiotherapy degrees: They had received training to carry out the assigned tasks. The procedure of taking anthropometrical measures took place in the school on the days that students had physical education classes. All collected data were entered in a preformatted spreadsheet and then checked for typing errors.

### 2.4. Statistical Analysis

Differences between groups were compared using Student’s *t*-test and a one-way ANOVA. For the purpose of analyzing factors associated with an optimal adherence to the MD according to the KIDMED score, the categories “very low” and “need to improve” were grouped, and a dependent binomial variable was generated in order to perform a multivariable logistic regression analysis of factors associated with following an optimal MD. The odds ratios (OR) were estimated, with 95% confidence intervals (95% CI) and a significance level of 5% (*p* ≤ 0.05). Among the factors possibly associated with adherence to the MD, we first considered dietary habits by including the presence of distractions (e.g., television) during main meals (i.e., breakfast, lunch, and dinner) and whether the child or adolescent was used to eating any of those meals alone. Second, we included a dichotomous variable indicating whether a child had the recommended adequate hours of sleep (i.e., the presence of healthy sleep habits) as a proxy for lifestyle. Third, we studied the association between MD adherence and physical activity by including the weekly frequency of vigorous and light physical activity. Fourth, to study socioeconomic aspects, we included whether the mother had been born outside of Catalonia, if the parents had accomplished a higher education level, and the FAS. Fifth, we explored what association the presence of vending machines at school as well as the availability of money at school had with the likelihood of following an optimal MD, if any. Furthermore, we included a set of confounding factors that had been well established in the literature to influence MD adherence: In particular, we considered school year, age, and body mass index [42,43].

## 3. Results

Participation rates were as follows. The absolute contact rate (the proportion of participants and nonparticipants to total eligible subjects) was 98.9% (98.6% in primary and 99.3% in secondary), a really high figure thanks to the collaboration of schoolteachers. The absolute cooperation rate (the proportion of participants to total subjects contacted) was 78.5% (80.8% in primary and 75.6% in secondary). The absolute response rate (the proportion of participants to total eligible subjects) was 77.6% (79.6% in primary and 75.1% in secondary). Table 1 contains the sample characteristics. Of the whole sample, 47% were male (46% of primary school students and 48% of secondary school students). Participants’ ages were between 6 and 18 years, with a mean age of 8.7 years for primary school students, whereas according to local statistical data the average age for the entire population of primary school students from Mataró is 8.4 years and 14.6 years for secondary school students [34], while the average for the entire population of secondary school students is 14.1 years. Regarding anthropometric characteristics, in primary school children, both boys and girls had a mean height of 1.32 m and a mean weight of 31.8 kg. The mean height and weight for secondary school girls was 1.59 m and 53.7 kg, respectively, while for boys, mean height was 1.67 m and mean weight was 56.3 kg: These differences were statistically significant.

The mean BMI values were 17.7 for primary school students and 20.9 for secondary school students. Fourteen percent of primary school children had *z*-score values above two, while only 1% had values below two. In secondary school students, these percentages were 5% and 1%, respectively. As much as 38% of the children and adolescents studied did not meet the minimum hours of sleep recommended by the World Health Organization (WHO). The percentage of fathers with higher education was 23% in the primary school group and 21% in the secondary school group, with no statistically significant differences between the two groups. For mothers, these values were 27% (primary) and 20% (secondary), which represented a statistically significant difference. The percentage of mothers born outside the region of Catalonia was 25% for mothers of primary school children, but this rose to 39% in mothers of secondary school children: The difference between these percentages was statistically significant. According to Mataró statistical data, the percentage of inhabitants born out of Catalonia is 39.1% [34].

**Figure 1:**
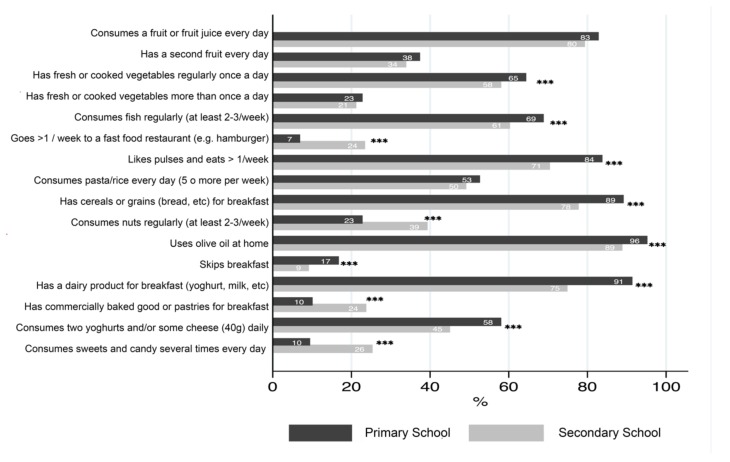
Responses to the questions on the Mediterranean Diet Quality Index for Children and Adolescents (KIDMED index) questionnaire showing the percentage of respondents who gave affirmative answers to each of the 16 questions on the KIDMED questionnaire. *** *p* < 0.01 using one-way ANOVA.

Figure 1 shows the results from the answers to the questions in the KIDMED questionnaire. Although there was a similar trend in both primary and secondary school groups, the secondary school group appeared to have a worse adherence to the MD. As can be seen in Figure 1, this was due mainly to the substantial percentage of adolescents who consumed fast food (24%), processed baked goods (24%), or sweets (26%) every day.

In addition, the results showed a low intake of fruit, vegetables, and nuts in children and adolescents. Furthermore, only 38% of primary school students and 34% of secondary school students reported eating a second portion of fruit per day. Likewise, only 23% of children and 21% of adolescents reported eating raw or cooked vegetables more than once per day. The regular consumption of nuts was only 23% in primary school students and 39% for secondary school students.

There was a statistically significant difference in the KIDMED index between primary school girls and secondary school girls, and also between boys and girls in primary and secondary school (Table 2). The difference between the KIDMED index for all primary school students (7.4 ± 0.1) and all secondary school students (6.3 ± 0.1) was statistically significant. However, no significant difference was found between sexes inside each group (primary or secondary school).

Table 2 shows the distribution by educational level and sex of the participants into three categories of degree of adherence to the MD, according to the score obtained on the KIDMED index. Without differentiating for sex, the percentage of students who did not have an optimal MD was 59% (51% of primary school students, 70% of secondary school students). Secondary school students had the highest rates of low index scores, as up to 11% of them had a very low-quality MD, while in primary school students, only 3% of children were classified as such.

**Figure 2:**
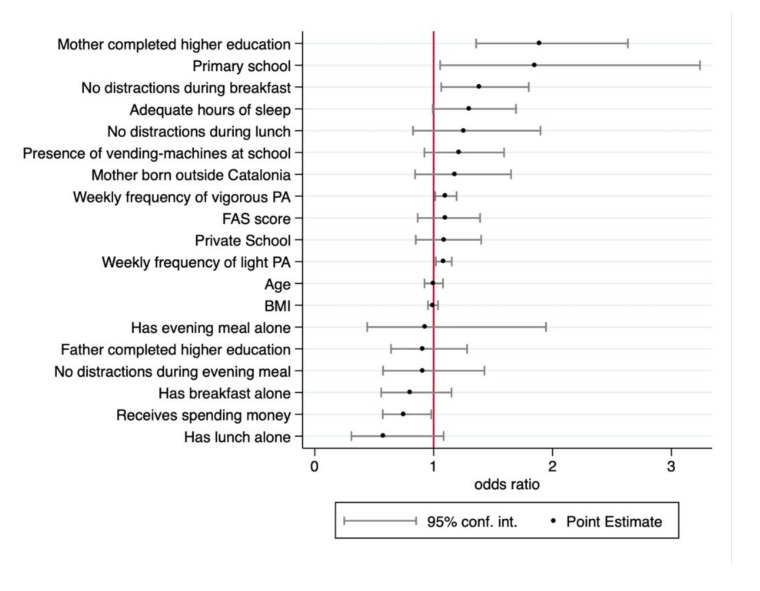
Results of the logistical model for factors associated with optimal adherence to the Mediterranean diet (MD). PA = physical activity; FAS = Family Affluence Scale; BMI: body mass index. The model includes Wald χ2 (17) = 91.91 (*p*-value < 0.001).

Figure 2 shows the results of the point estimates for the logistic regression, with the ORs presented in order of magnitude. As explained above, for this logistic model, the variable *KIDMED score* was recodified into two categories: Suboptimal (low and moderate adherence to MD) and optimal. Four factors stood out as being positively predictive of following an optimal MD. First, we observed that children and adolescents whose mothers had completed higher level education were more likely to have optimal adherence to a MD (OR = 1.89; 95% CI: 1.35–2.63). It was also interesting to note that, according to the data obtained in the questionnaire, the mother was responsible for the children’s food intake in 66% of primary school students and in 58% of secondary school students, while both mother and father were jointly responsible in 23% and 30% of cases, respectively. Second, with an OR of 1.84, boys and girls from primary school levels were more likely to follow an optimal MD than secondary school students, but with a really wide confidence interval (95% CI: 1.05–3.23). Third, the absence of distractions during breakfast was also a good predictor of optimal adherence to a MD (OR = 1.39; 95% CI: 1.06–1.81).

The last factor that appeared to be positively predictive of optimal adherence to a MD was doing regular physical activity. This variable was divided into two categories: (i) Vigorous, which involves at least 20 min of one of the sports of basketball, football, jogging, gymnastics, aerobics, or physical education class; and (ii) light, which includes walking or riding a bike continuously for at least 30 min, including when the activity is done on the way to school. Both vigorous and light activity were positively associated with optimal adherence to a MD: The OR for the vigorous activity category was 1.09 (95% CI: 1.01–1.19), and for the light category it was 1.08 (95% CI: 1.01–1.15).

The variable “hours of sleep” was divided into two categories (adequate or not adequate) using WHO recommendations (minimum of 10 h for children 6–13 years old and 8 h for adolescents). It should be noted that although hours of sleep did not have a statistically significant association with adherence to a MD, the data indicated some trend in this direction (OR = 1.29; 95% CI: 0.99–1.69; *p* = 0.05).

Regarding the factors negatively associated with optimal MD adherence, the availability of money to buy food during the school day was the only variable that was statistically significant, with an OR of 0.74 (95% CI: 0.57–0.97). Other factors studied, such as the presence of distractions when eating lunch or the evening meal and eating meals alone, had negative OR values, but these were not statistically significant.

Last, we must point out that there were no statistically significant associations between an optimal MD pattern and the factors of private school, age, BMI, FAS, mother’s country of origin, or father’s education level.

In order to confirm that overadjustment was not committed, an analysis was performed by running the model variable by variable without adjusting for other variables. This analysis confirmed that both significance and magnitude were consistent with the results of our model, so overadjustment was not present in our results. Thus, we can confirm that our results were robust under different modeling approaches.

## 4. Discussion

The results for the Mataró school-age population studied confirm the need to improve adherence to the MD pattern in this population: 48.2% of primary school students and 59.2% of secondary school students scored “low” or “moderate” in the adherence categories (and therefore need to improve their diet for adherence to be optimal). The multivariate logistic regression analysis showed a more optimal adherence to a MD in students from primary school levels whose mothers had completed higher education, who did not have distractions at breakfast time, and who regularly did physical activity. The availability of spending money was associated with a lower degree of adherence to a MD.

These figures for MD adherence were similar to those that have been obtained in other previous studies conducted in Spain, which found that in children and young people (2–24 years), between 46.7% and 69.1% needed to improve MD adherence to be considered optimal, respectively [11,16]. Similar percentages have been found for this segment of the population in countries such as Italy, Greece, and Turkey [5,11,17,18,44]. However, there has been significant variability in the figures on MD adherence in children and adolescents in different countries and even within Spain [11,45].

In recent decades, the dietary pattern in Spanish child and adolescent populations has progressively moved away from the MD due to a reduction in the intake of some of the key foods in this dietary pattern, such as fruit, vegetables, legumes, and fish [16,36]. This decline may be due to multiple complex factors that can be grouped into individual determinants (biological factors, preferences, nutritional knowledge, and attitudes) or collective determinants (economic, social, and physical factors) [46,47]. These determinants can interact in different ways.

The logistic regression results identified factors associated with a low adherence to the MD. The mother’s education level, but not the father’s, was positively associated with the degree of adherence to the MD. These observations coincided with those reported in other studies [12,16,17,22,32], which suggests that decisions regarding the diet of children and adolescents are made mainly by the mother. Mothers with a high educational level can influence the choices their children make by means of the availability and accessibility of certain foods, as well as by being a role model for them. It is also possible that a higher academic status is associated with higher income and consequently greater availability of healthy foods [32]. However, in our case, we did not detect an association between the FAS index and adherence to the MD.

In a study by Serra et al. [16], the authors attributed lower KIDMED index values in lower socioeconomic and educational (mother’s education level) strata to a lower consumption of oil, rice, pasta, fruits, and vegetables, suggesting the need to rethink the idea that the MD is inexpensive. Some studies on the financial cost of a MD appear to have pointed to it as having a higher cost compared to the “Western” diet in Spain, a difference that could have become more pronounced during the recent global financial crisis (2008–2012), just when our study was conducted [24]. However, the data from our study showed that a low socioeconomic status, determined using the FAS index, was not associated with lower KIDMED index values. It is possible that an association was not detected due to the limited reliability of the scoring index used (i.e., the FAS index) [48]. A recent systematic analysis [45] showed that most studies (15 out of 20) have detected an association between socioeconomic status and adherence to the MD, although it should be pointed out that in most cases, parents’ education level was used as a proxy variable.

In line with other similar studies [16,33,49], we observed that primary school children had a higher degree of adherence to the MD than secondary school students. This could be explained by adult supervision and control over children’s diets, an influence that is progressively lost as an individual grows up. Some studies have shown that this parental control is associated with following healthy dietary habits and patterns [50,51,52].

The absence of distractions during mealtimes is also associated with a higher degree of adherence to a MD, although in our study a statistically significant difference was found only for breakfast. Recent studies have suggested that eating without distractions (such as television) has a positive association with a healthy lifestyle and reduces the risk of obesity [29,53]. It is possible that the absence of distractions may facilitate family supervision and the adoption of good dietary habits and may help avoid the negative effects of television advertising of food on dietary behavior in children and adolescents [46].

Regarding physical activity, our results were in line with the literature, showing a positive association between physical activity and other healthy lifestyle habits such as an adequate diet [20,25,26,27,28], as well as adherence to a MD [45]. However, we did not detect an increase in the degree of adherence to a MD with increased intensity of physical activity (vigorous vs light), as has been observed in other studies [44].

This study demonstrated that adherence to a MD was lower among adolescents and children who had money to spend at school. The availability of money is not a risk factor per se if there is no easily accessible unhealthy food. However, when this is available, children and adolescents can be influenced and may change their usual dietary habits. In fact, some studies have suggested that constant exposure to the offer of certain foods with a high calorie content or poor nutritional profile is positively associated with a deterioration in the nutritional state of children and young people [54,55,56]. As mentioned in the results, a reduction in MD adherence in adolescents was due in large part to an increase in the consumption of fast food, baked goods, and sweets. This offer of low-nutritional-value food is common in facilities and establishments that adolescents often use and can be found in the places where they carry out their daily activities: Fast food restaurants, cafes, convenience shops, places with vending machines, etc. [47]. The lower level of parental supervision due to adolescents’ progressive independence, as well as the influence of their circle of friends and advertisements of certain foods, may facilitate negative changes at that point in the quality of their diet [46,47]. Likewise, higher prices in certain foods is another determinant that might explain this lower MD adherence in adolescents [57].

Finally, as in other studies on the factors associated with adherence to a MD [45], we did not find a statistically significant association with control variables such as age, sex, body mass index, or parents’ country of origin. In our study, we observed a nonsignificant trend that children and adolescents getting fewer hours of sleep were less likely to have optimal adherence to the MD. This merits attention in future studies, as there is some existing evidence on the association between sleep duration and quality and diet [58], including the adherence to the MD in young adolescents [59]. In that sense, some studies have found an association between irregular eating, skipping breakfast, and lower intake of fruits and vegetables, all of them related to lower adherence to the MD. However, the relation between MD adherence and sleep duration remains unclear, and the mechanisms that explain such an association are likely to be multifactorial [60].

The design of this study did not allow us to establish causality for the significant associations studied. However, the concurrence with the results obtained in other studies highlights the interest in investigating the possible causality and mechanisms involved using appropriate designs. Another limitation of the study, with regard to the population older than 12 years, was the use of questionnaires completed by the adolescents themselves. Although a teacher was present and available to help, this could represent information bias, as we could not confirm the reported information with the participants’ parents. In addition, although the sampling design guaranteed the sample was representative of the city of Mataró, the results cannot be generalized to the Spanish population as a whole or to the rural population of Catalonia, because regional differences have been described in Spain as well as in rural versus urban populations [16]. Finally, this study lacked detailed information on factors related to socioeconomics, family history, and more objective evidence of the adherence to a MD, such as biochemical markers.

## 5. Conclusions

This study suggests that some factors (mother’s education level, distractions at breakfast time, physical activity, and the availability of spending money) are associated with the likelihood of optimal adherence to the MD.

In view of these results, the design and implementation of any intervention to promote healthy habits and lifestyles (e.g., educational interventions) among children and adolescents of school age should consider the participation of parents, especially those with lower education levels. Likewise, another central component is targeting the period of adolescence, as there appears to be a decline in MD adherence in this segment of the population. Schools could be a convenient environment for such interventions.

Future research should study more in-depth the possible causality between the factors studied and adherence to the MD, as well as the mechanisms explaining it. What is also worthy of attention is research into the influence of the community environment to determine the effect of the offer of food on the habits of children and particularly adolescents.

## Figures and Tables

**Table 1:** Description of the main characteristics of the sample of primary and secondary school groups according to sex ^1^.

	Primary			Secondary		
	Total (*n* = 668)	Male (*n* = 310)	Female (*n* = 358)	Total (*n* = 509)	Male (*n* = 246)	Female (*n* = 263)
	Mean ± SD	Mean ± SD	Mean ± SD	Mean ± SD	Mean ± SD	Mean ± SD
Age	8.7 ± 1.7	8.7 ± 1.7	8.6 ± 1.67	14.9 ± 1.9	15.0 ± 1.9	14.9 ± 1.8
Weight (kg)	31.8 ± 9.2	31.8 ± 9.0	31.8 ± 9.5	56.3 ± 12.5	59.1 ± 14.3 *	53.7 ± 9.9 *
Height (m)	1.32 ± 0.11	1.33 ± 0.11	1.31 ± 0.11	1.63 ± 0.09	1.67 ± 0.10 *	1.59 ± 0.07 *
BMI (kg/m^2^)	17.7 ± 2.9	17.6 ± 2.7	17.9 ± 3.1	20.9 ± 3.3	20.9 ± 3.5	20.9 ± 3.1
Adequate hours of sleep (%)	62.0	63.0	61.0	62.0	66.0	59.0
Mother completed higher education (%)	27.0 †	29.0 †	26.0	20.0 †	21.0 †	19.0
Father completed higher education (%)	23.0	26.0	20.0	21.0	21.0	21.0
Mother born outside Catalonia (%)	25.0 †	26.0 †	25.0 †	39.0 †	42.0 †	37.0 †
Low FAS (%)	4.7	4.5	4.8	2.8	2.0	3.4
Medium FAS (%)	64.1†	67.8 †	60.9 †	43.0 †	41.1†	44.9 †
High FAS (%)	31.2 †	27.7 †	34.3 †	54.2 †	56.9 †	51.7 †

^1^ For each variable, the groups with the same superscript present statistically significant differences (*p*-value < 0.05). BMI: body mass index; FAS: Family Affluence Scale. * Differences between sexes in each school group; † differences between school groups: total, male, and female.

**Table 2:** Mean scores on the KIDMED index and percentage distribution of the respondents in the three categories of adherence to the Mediterranean diet (MD) (low, moderate, optimal) in accordance with the score obtained on the KIDMED index.

	Primary		Secondary	
	Male (*n* = 310)	Female (*n* = 358)	Male (*n* = 246)	Female (*n* = 263)
	Mean ± SD	Mean ± SD	Mean ± SD	Mean ± SD
KIDMED score	7.4 ± 1.9 †	7.3 ± 1.9 †	6.3 ± 2.3 †	6.3 ± 2.0 †
Distribution by categories of the KIDMED score (%)				
LOW adherence	3.5	2.2	12.6	8.7
MODERATE adherence	45.5	50.8	54.1	64.3
OPTIMAL adherence	51.0	46.9	33.3	27.0

In all of the variables included in the table, the results obtained presented statistically significant differences between the school groups for each sex (*p*-value < 0.05) according to the Student’s *t*-test (KIDMED score) or the analysis of variance (categories of the KIDMED score). † Differences between school groups for males and females. We did not find any significant differences within gender for each school group.
